# Weight Loss Outcomes Among MyFitnessPal Users: Behavioral and Dietary Predictors of Success

**DOI:** 10.3390/nu18111766

**Published:** 2026-05-30

**Authors:** Bingling Wang, Lucia Y. Chen, Hwan Cho, Jieping Yang, Chi-Hong Tseng, Zhaoping Li

**Affiliations:** 1 Statistics & Data Science, UCLA, 8125 Math Sciences Bldg. Box 951554, Los Angeles, CA 90095, USA; bingling.wang@stat.ucla.edu (B.W.); luciachen@mednet.ucla.edu (L.Y.C.); hwancho19@ucla.edu (H.C.); 2 Center for Human Nutrition, David Geffen School of Medicine at UCLA, 1000 Veteran Ave, Los Angeles, CA 90095, USA; jiepingyang@mednet.ucla.edu; 3 Department of Medicine Statistics Core, David Geffen School of Medicine at UCLA, 1100 Glendon Avenue, 8th Floor, Los Angeles, CA 90024, USA; ctseng@mednet.ucla.edu

**Keywords:** MyFitnessPal, digital health, weight loss, dietary intake, obesity

## Abstract

Background/Objectives: This study aimed to evaluate the associations between MyFitnessPal usage, a widely used smartphone application, and weight management outcomes among U.S. adults, and to examine factors associated with clinically significant weight loss. Methods: Data from 1359 users who set weight loss goals and maintained consistent app engagement over 120 days were analyzed. Demographic characteristics, app engagement patterns, and dietary intake were compared between users who did and did not achieve clinically significant weight loss. Results: Nearly half (48.5%) of participants achieved clinically significant weight loss (≥5% of body weight), with success rates varying notably across age groups. App engagement was strongly associated with weight-loss success, with users maintaining detailed food logs achieving substantially higher success rates than the full cohort. Multivariable logistic regression identified daily app engagement as the significant predictor of weight-loss success, followed by lower carbohydrate intake, higher initial weight, and younger age. Conclusions: These findings indicate that greater engagement with MyFitnessPal is associated with favorable weight management outcomes and suggest that digital health platforms may be a scalable intervention for obesity prevention and treatment.

## 1. Introduction

The global obesity epidemic remains a significant public health challenge, with over 1 billion people worldwide currently living with obesity [1]. Globally, the prevalence of obesity has more than tripled between 1975 and 2022, with adult obesity rates nearly tripling among women (6.6% to 18.5%) and quadrupling in men (3% to 14.0%) [2]. Obesity substantially increases the risk of chronic diseases, including type 2 diabetes, cardiovascular disease, and certain cancers, and contributes to high healthcare costs, functional limitations, and reduced quality of life [3]. Although traditional weight management interventions, such as in-person counseling and structured clinical programs, can be effective, their broad implementation is often limited by high costs, limited access, and poor long-term adherence. Consequently, digital health tools, particularly smartphone-based applications, have emerged as promising, scalable approaches for supporting behavior change and weight management.

Smartphone applications enable users to self-monitor dietary intake and physical activity through real-time tracking, personalized goal setting, and automated feedback. Growing evidence supports the effectiveness of these tools for weight management. Studies of app-based interventions have shown that consistent self-monitoring through mobile applications is associated with greater weight loss success [4]. Burke et al. found that participants using personal digital assistants (PDAs) with dietary software maintained higher adherence to self-monitoring than those using paper records, and adding daily tailored feedback messages further enhanced weight loss outcomes [5]. Large-scale observational data from commercial apps and wearable devices have provided opportunities to study weight management behaviors in real-world settings, complementing controlled trials and identifying predictors of successful outcomes at the population level [6].

MyFitnessPal is one of the most widely used smartphone applications for weight management worldwide, offering a comprehensive platform for tracking dietary intake, physical activity, and body weight. Key features include a large food database, barcode scanning, automatic calorie and nutrient calculations, and progress visualization tools. Although prior studies have examined app-based weight loss, fewer have incorporated detailed dietary intake analyses using real-world app data [7,8]. Validation studies show that MyFitnessPal provides reasonably accurate and efficient estimates of total energy intake, macronutrients, sugar, and fiber [9].

This study aimed to evaluate weight-management behaviors, including dietary behavior, among MyFitnessPal users over 120 days. Using behavioral and demographic data from a large cohort, we examined app engagement patterns and factors associated with successful weight management. Specifically, we sought to: (1) assess overall weight loss and the proportion achieving clinically significant loss (≥5%); (2) compare demographic and behavioral characteristics between users with or without successful weight loss; and (3) identify independent predictors of weight-loss success through multivariable modeling.

## 2. Methods

### 2.1. Study Design and Population

This retrospective observational study analyzed anonymized data from U.S.-based MyFitnessPal users who set a weight loss goal during a 120-day observation period beginning January 2022. All data were fully de-identified prior to author access, and no personally identifiable information was available, nor was re-identification possible. MyFitnessPal is a mobile health application that supports weight management through self-monitoring of dietary intake, physical activity, and body weight. The app provides a large food database, barcode scanning, automated calorie and nutrient calculations, and progress visualization. Upon account setup, users enter weight, height, and weight-related goals, and receive individualized calorie recommendations. Daily logs of food consumption and exercise generate reports on weight trends, calorie balances, and nutrient intake, with premium subscribers having access to advanced features such as detailed macronutrient breakdowns and more personalized guidance. Users consented to data collection and use through acceptance of the platform’s terms of service and privacy policy.

Participants were included if they were U.S.-based, set a weight loss goal at account creation, and logged weight measurements for at least four consecutive weeks, demonstrating sustained engagement. Users with missing or implausible values for weight, body mass index (BMI), or age were excluded. The final analytic cohort comprised 1359 participants.

### 2.2. Variables and Measures

Demographic variables included age, gender, initial body weight, BMI, and subscription status. Self-monitoring behaviors were assessed using app usage metrics, including frequency of weight logging, dietary logging, water intake logging, meal scanning, barcode scanning, and overall login frequency. A composite engagement metric was calculated by summing these six activity types, including frequency of weight logging, dietary logging, water intake logging, meal scanning, barcode scanning, and overall login frequency, to produce total daily app logs, serving as a proxy for overall engagement.

Nutritional intake data were obtained through the app’s food database and barcode scanning functionality. For users logging food on at least 60 days of the 120-day period, average daily intake was calculated for 12 key dietary components: total calories, protein, carbohydrates, total fat, polyunsaturated fat, trans fat, fiber, sugar, sodium, potassium, calcium, and iron. This threshold balanced data completeness with statistical power.

### 2.3. Outcome Definition

Participants were classified as Responders if they lost ≥5% of their initial body weight during the study period and as Non-Responders if weight loss was <5%. This threshold, supported by NIH guidelines [10] and clinical practice recommendations [11], represents clinically meaningful weight loss associated with improvements in cardiometabolic outcomes in individuals with overweight or obesity. A 5% weight loss is considered as clinically meaningful even in normal-weight individuals, yielding improvements in metabolic, cardiovascular, and inflammatory markers, with benefits comparable in magnitude to those observed in individuals with obesity [12].

### 2.4. Statistical Analysis

Exploratory data analysis was conducted to assess data integrity, identify missing values, and examine distributional properties of key variables. Descriptive statistics were calculated for demographic characteristics, baseline weight status, engagement patterns, and nutritional intake, stratified by outcome group (Responders vs. Non-Responders) with comparative tests to evaluate group differences.

Regression analysis was performed on the subsample of users who logged food on at least 60 days out of the 120-day period. Correlation analyses examined relationships among nutrient variables to identify potential multicollinearity prior to modeling. Logistic regression was used to identify independent predictors of weight loss, with outcome (Responder vs. Non-Responder) as the dependent variable. The initial model included 16 candidate predictors: initial weight, age, gender, protein, fiber, total fat, sodium, carbohydrates, sugar, calcium, potassium, iron, trans fat, polyunsaturated fat, subscription status (premium vs. non-premium), and average daily app logs. Total calories were excluded due to high correlations (r = 0.56–0.75) with macronutrients, and the Variance Inflation Factor (VIF) analysis confirmed acceptable multicollinearity (all VIF < 3). Dietary variables were scaled by a factor of 10 to avoid trivial or overly precise point estimates. Results were also stratified by BMI group (normal weight vs. overweight/obese), and odds ratios (ORs) with 95% confidence intervals (CIs) are presented, as well as Akaike Information Criteria (AIC) for assessing model fit.

Lasso-penalized logistic regression was conducted to identify a parsimonious set of predictors. The set of 16 predictors was entered into a LASSO model, and five-fold cross-validation was used to select the most regularized model whose performance remained within one standard error of the optimal model. Predictors that retained nonzero penalized coefficients were then entered into a subsequent multivariate logistic regression model to obtain ORs and 95% CIs. Area under the curve (AUC) is presented, and calibration was examined using a calibration plot.

Influential observations were assessed using DFBETAs. Complete case analysis was performed. All statistical tests were two-tailed with α = 0.05, and analyses were conducted in R v4.5.3 statistical software.

## 3. Results

### 3.1. Participant Characteristics and Overall Success Rate

A total of 1359 participants met the inclusion criteria and comprised the final analytic cohort. The sample was relatively balanced by gender, with 704 females (51.8%) and 655 males (48.2%) participants. Participants ranged in age from 18 to 83 years, with a distribution centered around middle age (mean age = 41 years). The largest proportion fell in their thirties (27.2%, *n* = 370), followed by participants in their forties (21.0%, *n* = 286) and twenties (18.8%, *n* = 255). Smaller proportions were observed among those in their fifties (18.5%, *n* = 251), those aged 60 years or older (10.4%, *n* = 42), and those aged 18–19 years (4.1%, *n* = 55).

Initial body weights ranged from 97 to 430 pounds, with a mean of 195.7 lbs (SD = 46.0), corresponding to a mean body mass index (BMI) of 28.1 (SD = 5.5). Overall, 69% of participants (*n* = 936) had BMI values indicating overweight or obesity (BMI ≥ 25), while 31% (*n* = 423) fell within the healthy range (BMI 18.5–24.9). Stratified by gender, a higher proportion of male participants (77%) were above the healthy BMI range compared to female participants (61.5%) (Table 1).

With respect to weight-loss outcomes, 659 (48.5%) achieved clinically significant weight loss (≥5%) within the 120-day study period and were classified as Responders, while 700 (51.5%) achieved <5% weight loss and were classified as Non-Responders.

### 3.2. Success Rates by Demographic Characteristics

**Figure 1:**
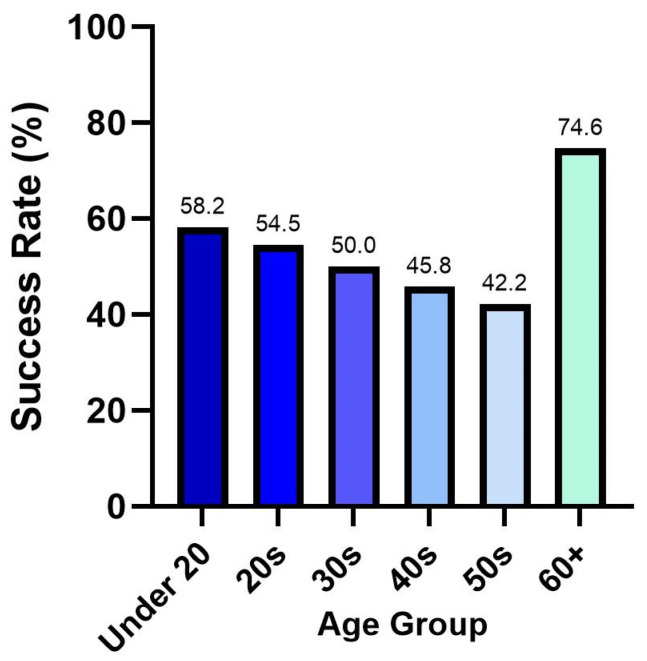
Success Rates Across Age Groups.

Success rates showed modest gender-based variation. Male participants achieved a success rate of 51.0%, compared with 46.1% among females—a 4.9 percentage point difference that was not statistically significant (χ^2^ = 3.21, *p* = 0.073). Age-related differences were more pronounced (Figure 1). Success rates declined steadily from younger adulthood through midlife, with participants under 20 years achieving a success rate of 58.2%, followed by those in their twenties 54.5%, thirties 50%, and forties 45.8%. Participants in their fifties exhibited the lowest success rate at 42.2%. In contrast, participants aged 60 years and older showed the highest success rate at 74.6%, substantially exceeding all other age groups. Collectively, these findings reveal a U-shaped pattern in success across the lifespan.

### 3.3. Impact of Subscription Status and Engagement Behavior on Success Rates

**Figure 2:**
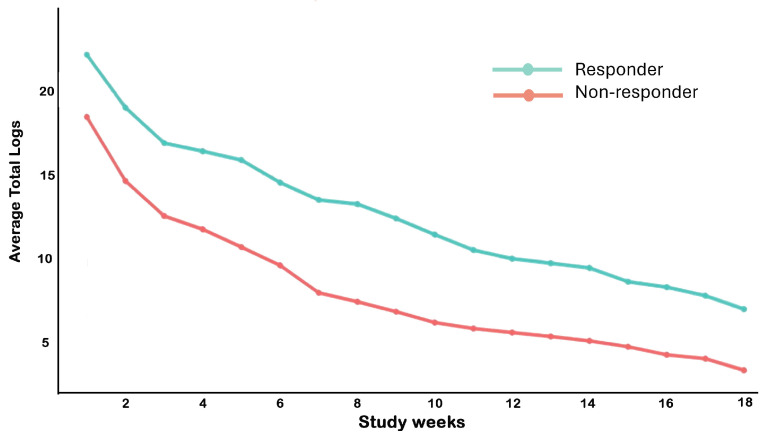
Weekly App Engagement Over Time.

Among 1359 participants, 416 (30.6%) held trial subscriptions, 284 (20.9%) had paid subscriptions, and 659 (48.5%) were non-subscribed users. Success rates did not differ significantly between subscribed (trial and paid) and non-subscribed users (50.0% vs. 47.8%, χ^2^ = 0.46, *p* = 0.496). Among trial users, 68.3% converted to paid subscriptions during the study period. Weekly average app engagement over the 120-day study period is shown in Figure 2. Engagement declined over time in both Responders and Non-Responders; however, Responders consistently demonstrated higher activity levels throughout the study. During the first week, Responders recorded an average of approximately 22 daily logs, compared with 18 daily logs for Non-Responders. This engagement gap persisted through the end of the study, with Responders averaging 7 daily logs versus 3.5 for Non-Responders (*p* < 2.2 ×10^−16^).

**Figure 3:**
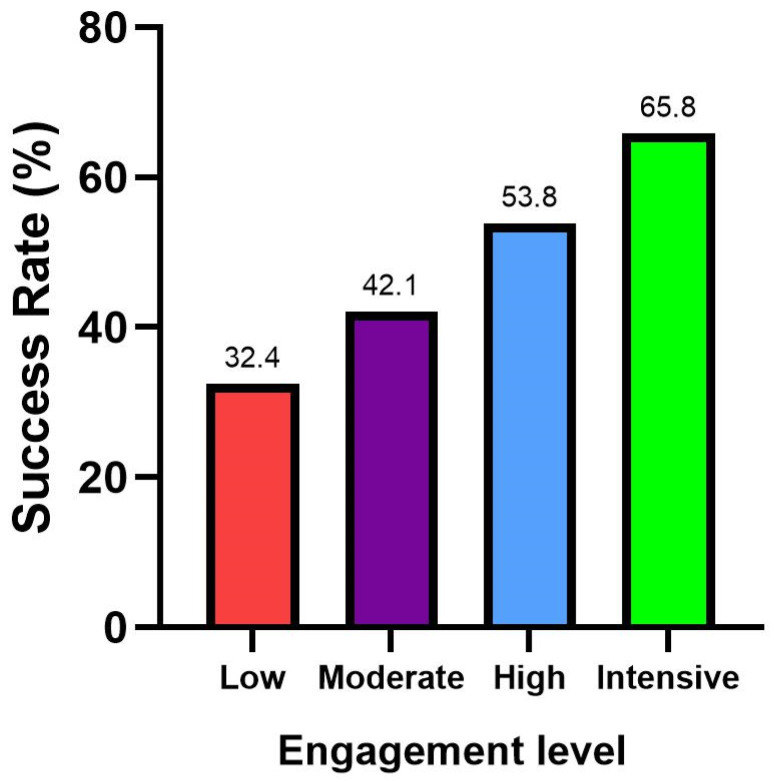
Success Rate by User Engagement Level.

To further examine the association between app engagement and weight loss success, a composite engagement metric was created by combining six types of daily activities: food logging, water logging, weight logging, meals scanning, barcode scanning, and app logins. Participants were divided into quartiles representing Low, Moderate, High, and Intensive Engagement levels (Table 2). Mean daily activity increased stepwise across quartiles, ranging from 1.89 logs per day in the Low Engagement group to 23.1 logs per day in the Intensive Engagement group. Weight loss success increased progressively across engagement levels. Success rates were 32.4% in the Low Engagement group, 41.8% in the Moderate group, 53.4% in the High group, and 65.8% in the Intensive group. A Cochran–Armitage test for trend confirmed a highly significant positive association between engagement level and success rate (χ^2^ = 85.34, df = 1, *p* < 2.2 × 10^−16^) (Figure 3).

### 3.4. Nutritional Intake Patterns on Success Rates

Of the 1359 participants, 647 met the predefined 60-day food logging threshold, had complete data, and were included in the nutritional analysis. This subsample consisted of 389 Responders and 258 Non-Responders, corresponding to an overall success rate of 60.1% among these selected, relatively adherent users meeting the threshold. Compared with Non-Responders, Responders consumed lower carbohydrates (median: 125 vs. 138 g/day, *p* = 0.001) and sugar (median: 41 vs. 44 g/day, *p* = 0.007). Protein intake was modestly higher among Responders (median: 76 vs. 71 g/day) but did not reach statistical significance (*p* = 0.13). Total caloric intake, total fat, fiber, and micronutrients, including sodium, potassium, calcium, and iron, were similar between groups. Full daily nutrient intake values are presented in Table 3.

### 3.5. Predictors of Weight Loss Success: Logistic Regression Analysis Among Participants Meeting the 60-Day Food-Logging Threshold

Dietary, behavioral, and demographic variables were further evaluated using multivariable logistic regression among participants who met the predefined 60-day food-logging threshold, to identify factors associated with weight loss success within this consistently logging population (n = 647). The full logistic regression model, including 16 predictors (AIC = 813.09), identified four significant factors associated with weight loss success: younger age (*p* = 0.012), higher initial weight (*p* < 0.001), lower carbohydrate intake (*p* = 0.015), and higher average daily app logging frequency (*p* < 0.001). Protein intake showed borderline significance (*p* = 0.063). All other dietary variables and subscription status (premium vs. non-premium) were not significantly associated with success. Interestingly, age showed a negative linear relationship with odds of weight loss success in this subcohort, and the U-shaped pattern seen in the full cohort was not observed here.

This analysis was also stratified by BMI category (normal weight: BMI < 25; overweight/obese: BMI ≥ 25). Among overweight/obese participants, younger age (*p* = 0.002), higher initial weight (*p* < 0.001), lower sugar intake (*p* = 0.024), and greater average daily app logging frequency (*p* < 0.001) remained significant predictors of weight loss success. In contrast, among normal-weight participants, male gender (*p* = 0.031), higher initial weight (*p* < 0.001), lower protein intake (*p* < 0.001), lower carbohydrate intake (*p* = 0.009), and higher average daily app logging frequency (*p* = 0.001) were significantly associated with weight loss success.

In addition, we performed variable selection using LASSO to create a parsimonious model. Three predictors retained nonzero coefficients and were entered into a final parsimonious logistic regression model: initial weight, carbohydrates, and average daily app logs. The AIC for this parsimonious model was 805.11, indicating slight improvement over the full model (ΔAIC = −7.89), and the area under the curve was 0.70, representing moderate predictive performance. Internal bootstrap calibration demonstrated good agreement between predicted and observed response probabilities (Supplementary Figure S1). Table 4 presents the results of the final parsimonious model. We also repeated this process stratifying by baseline BMI category (Supplementary Table S1).

Outlier assessment using DFBETAs revealed several moderately influential observations. Sensitivity analysis excluding these observations did not yield materially different model estimates.

## 4. Discussion

### 4.1. Principal Findings

This large-scale observational study of 1359 MyFitnessPal users demonstrated that nearly half achieved clinically significant weight loss (≥5%) within 120 days. Weight-loss success was strongly associated with sustained app engagement, with users in the highest engagement quartile more than twice as likely to achieve their goals compared to those in the lowest quartile. Notably, success rates varied considerably across age groups, revealing a U-shaped pattern with peak success among older adults and lowest success among middle-aged adults. Multivariable analysis identified greater app engagement, lower carbohydrate intake, male gender, and younger age among users who maintained adequate food logging as significant predictors of success. Multiple meta-analyses have consistently shown that mobile health applications are associated with improved weight management outcomes [13,14]. Apps that incorporate multiple behavior change techniques—most commonly self-monitoring, goal setting, and action planning—tend to perform better than those with a single behavioral focus. Interventions that include eight or more behavior change techniques and integrate dietary monitoring with physical activity components demonstrate more favorable outcomes [15]. Consistent with these findings, our results highlight key behavioral and dietary factors associated with successful weight management in real-world digital health settings. However, these findings should be interpreted with caution as the initial inclusion criterion of at least 4 weeks of weight logging, along with the minimum 60-day food logging threshold used in subsequent multivariable modeling, likely selected for a more highly engaged subgroup. This may have inflated the observed success rates and limited the generalizability of the findings.

### 4.2. App Engagement: The Dominant Predictor of Weight Loss Success

Consistent with prevailing evidence, greater engagement with weight-loss applications is strongly associated with improved weight-loss outcomes [16]. In our analyses, App engagement emerged as a significant predictor of weight-loss success across all analyses. Descriptively, Responders maintained consistently higher engagement than Non-Responders throughout the 120-day study period, starting at 22 versus 18 daily logs in week 1 and declining to 7 versus 3.5 logs at the end of the study. Although engagement declined over time in both groups—consistent with typical attrition observed in digital health interventions—Responders sustained higher engagement levels throughout. This persistent difference in engagement, rather than initial motivation alone, distinguished successful from unsuccessful users. Each additional daily app interaction increased the odds of achieving ≥5% weight loss by 8%. The cumulative effect is substantial: users averaging 15 daily logs had more than twice the odds of success compared to users averaging 5 daily logs.

These findings have important implications for app design and intervention strategies. Features that promote sustained engagement may enhance intervention effectiveness. The temporal pattern showing gradual decline in both groups suggests that maintaining engagement beyond initial motivation is a critical challenge. Understanding barriers to sustained use among less engaged users may help improve long-term outcomes.

### 4.3. Dietary Patterns and Weight Loss Success

Among selected participants who consistently logged food and met the predefined 60-day food-logging threshold, lower recorded carbohydrate intake was associated with higher odds of weight-loss success (OR = 0.94, *p* = 0.015), independent of total caloric intake. While the per-gram effect was small, larger differences in recorded carbohydrate intake (e.g., ~50 g/day) corresponded to higher odds of success. However, given the reliance on self-reported app-based intake data and the fact that food logging itself is part of the engagement profile and likely correlated with both dietary estimates and weight-loss success, these findings should be interpreted with caution. Protein intake was similar between Responders and non-responders in the full cohort (76 vs. 71 g/day; *p* = 0.13). However, in the highly adherent subcohort meeting the minimum 60-day food logging threshold, protein intake demonstrated a decreasing trend and showed a borderline statistical significance in the multivariable model (OR = 0.93, *p* = 0.0639). These findings suggest that, within this analysis, the independent contribution of protein intake to weight loss outcomes is smaller than that of carbohydrate intake and should be further evaluated in future studies. These findings indicate the importance of macronutrient composition in digital weight management and the potential for apps to improve outcomes by providing guidance on dietary quality, carbohydrate reduction, and balanced meal planning.

### 4.4. Demographic Factors and Weight Loss Success

Age remained as a significant predictor after controlling for behavioral, initial weight, and dietary factors in the full cohort as well as in overweight/obese subgroup, while gender remained significant in healthy weight subgroup. Prior evidence from mobile intervention studies shows inconsistent gender related effects [17]. In the full cohort, success rates appeared higher in males than in females without statistical significance (*p* = 0.073). In the selected highly adherent subcohort, a significant difference was observed only among participants with a healthy BMI, but not among those who were overweight or obese. Specifically, males of healthy weight had a significantly lower success rate than females (OR = 0.33, *p* = 0.031). The wider confidence interval for gender reflects greater variability compared to behavioral and dietary predictors, likely due to the categorical nature of the variable and diverse individual responses within each gender.

In the full cohort, younger participants aged 30 and under demonstrated relatively high success rates, all exceeding 50%. Success rates declined progressively through middle age, from 50% in the thirties to 45.8% in the forties, reaching the lowest point of 42.2% among participants in their fifties. In striking contrast, participants aged 60 years and older achieved the highest success rate at 74.6%, markedly exceeding all other age groups and representing a sharp reversal of the middle-age decline. Although younger age has been identified as the most consistent predictor of greater participation and uptake of mobile interventions targeting weight-related behaviors [18], the observed U-shaped pattern deviates from this expectation and warrants further investigation. In the logistic regression limited to the subsample meeting the 60-day food logging threshold (n = 647), this U-shaped relationship between age and success was not observed. Instead, age demonstrated a modest negative linear association with weight loss success (OR = 0.98 per year, *p* = 0.0124), indicating slightly lower odds of success with increasing age among this motivated subgroup. This negative linear trend is consistent with the declining success rates observed through middle age, highlighting that younger participants in the engaged subsample were more likely to achieve weight loss success.

### 4.5. Public Health Implications

These findings have several implications for obesity management. Digital self-monitoring tools such as MyFitnessPal may serve as a scalable, accessible platform that complements traditional weight management approaches. In this study, nearly half of users achieved clinically significant weight loss; however, in the absence of a control group, it is not possible to determine whether app use directly contributed to weight loss. Moreover, analyses conducted within a more engaged subcohort showed that participants who maintained consistent food logging demonstrated higher success rates than those with lower engagement (60.1% vs. 48.5%), suggesting an association between sustained engagement and weight-loss outcomes. Variation in success across age groups further suggests that digital platforms may benefit from age-tailored content and support strategies to better align with users’ needs and preferences. Interestingly, when the analysis was stratified by BMI category, age emerged as a significant predictor of weight loss among overweight/obese participants, but not among those with a healthy weight. In contrast, gender was a significant predictor among participants with a healthy BMI, but not among those who were overweight or obese. These findings highlight that predictors of weight loss are not uniform across all patients—they vary by baseline weight status. In addition, dietary quality, particularly lower carbohydrate intake, was associated with weight loss success independent of total calories, highlighting the value of guidance beyond simple calorie counting. Overall, these findings reinforce that sustained engagement and dietary quality are important factors associated with weight management outcomes among real-world app users, while underscoring the need for cautious interpretation and future studies to establish causal relationships.

### 4.6. Limitations

Several limitations should be considered when interpreting these findings. First, reliance on self-reported data may introduce bias, as users could under-report food intake or selectively log healthier eating occasions. This may affect dietary intake estimates and potentially inflate apparent success among users who log more selectively. Although the large sample size and requirement for sustained engagement (≥4 weeks of weight logging) may partially mitigate random reporting error, this inclusion criterion likely selected for more highly engaged users, which may have further inflated observed success rates and limited the generalizability of the findings. Second, the 120-day observation period, while longer than many app-based studies, limits insights into long-term weight maintenance. Given the common occurrence of weight regain, future research should examine outcomes over extended periods. Third, although multivariable models controlled for measured confounders, unmeasured factors—such as baseline motivation, health literacy, social support, and participation in concurrent interventions—may influence both engagement and weight loss success. MyFitnessPal does not include a built-in feature for documenting this information. Randomized trials would be needed to establish causal relationships definitively. Finally, approximately one-third of the sample had a BMI in the normal or healthy-weight range, a group that potentially differs from individuals with this information. These users were included because they explicitly set weight-loss goals, and similar populations have been examined in prior weight loss research [12]; however, their inclusion may still limit generalizability. Future studies with larger and more balanced samples across BMI categories are needed to clarify whether engagement patterns and weight-loss outcomes differ between normal-weight and overweight/obese users.

### 4.7. Future Directions

Future research should address several key priorities. Longitudinal studies with follow-up periods of 12 months or longer are needed to assess sustained weight loss and identify predictors of long-term maintenance. Randomized controlled trials comparing app features and engagement strategies would clarify which elements most effectively support success and minimize attrition. Studies should also explore personalized approaches based on user characteristics to determine whether tailored interventions improve outcomes compared to standardized programs. Finally, implementation research is necessary to evaluate how digital tools can be optimally integrated into clinical practice and public health initiatives, including the roles of healthcare providers and sustainable reimbursement models.

## 5. Conclusions

This study examined real-world patterns of weight management among MyFitnessPal users and found that nearly half of engaged users achieved clinically significant weight loss within 120 days. Higher levels of sustained app engagement were strongly associated with weight loss outcomes. In addition, lower carbohydrate intake was identified as an independent dietary predictor, highlighting the role of macronutrient quality alongside behavioral engagement. Weight loss outcomes also varied across demographic groups, revealing notable patterns across age ranges. These findings underscore the potential of digital health tools as scalable, accessible interventions for weight management and emphasize the importance of strategies that promote ongoing engagement and comprehensive dietary tracking to maximize impact.

## Figures and Tables

**Table 1:** Baseline Characteristics of the Study Participants.

Characteristics	Statistics	
Gender	Female: 704 (51.8%)	
	Male: 655 (48.2%)	
Age (years)	Range: 18~83 Mean Age: 41	(18, 20): 4.1% (55 users)
		(20, 30): 18.8% (255 users)
		(30, 40): 27.2% (370 users)
		(40, 50): 21.0% (286 users)
		(50, 60): 18.5% (251 users)
		60+: 10.4% (142 users)
Weight (lbs.)	Range: 97~430 Mean (SD): 195.7 (46.0)	
BMI	Range: 18.5~56.8 Mean (SD): 28.1 (5.5)	Normal (18.5–24.9): 423 (31%) Overweight (>25): 936 (69%)
Subscription status	Trial: 416 (30.6%) *	
	Subscribed: 284 (20.9%) *	
	Non-subscribed (48.5%)	

* Conversion (trial → subscribed): 68.3%.

**Table 2:** Average Daily Logging Activity by User Engagement Level.

Engagement Level	Mean (SD)
Low Engagement	1.89 (1.00)
Moderate Engagement	5.75 (1.25)
High Engagement	11.3 (1.90)
Intensive Engagement	23.1 (7.91)

**Table 3:** Daily Nutrient Intake by Weight Loss Outcome. Values presented as median (IQR).

Nutrient	Full Cohort (*n* = 647)	Non-Responders (*n* = 258)	Responders (*n* = 389)
Total Calories (kcal/day)	1404 (1164–1706)	1406 (1208–1747)	1403 (1142–1676)
Carbohydrates (g/day)	130 (101–162)	138 (108–170)	125 (99–156)
Protein (g/day)	74 (55–97)	71 (53–95)	76 (57–98)
Total Fat (g/day)	54 (42–67)	54 (44–68)	53 (42–67)
Polyunsaturated Fat (g/day)	3.35 (2.51–4.47)	3.34 (2.53–4.39)	3.35 (2.44–4.47)
Trans Fat (g/day)	0.66 (0.33–1.28)	0.71 (0.39–1.40)	0.64 (0.31–1.16)
Fiber (g/day)	15 (11–20)	15 (11–20)	15 (11–21)
Sugar (g/day)	42 (30–54)	44 (34–56)	41 (29–53)
Sodium (mg/day)	1968 (1568–2475)	1897 (1501–2349)	2028 (1581–2519)
Potassium (mg/day)	981 (707–1353)	944 (712–1285)	1004 (705–1413)
Calcium (mg/day)	73 (51–103)	72 (48–101)	74 (52–103)
Iron (mg/day)	36 (27–50)	35 (28–50)	36 (27–50)

**Table 4:** Final Parsimonious Model Predicting Weight Loss Success.

Variable	Coefficient (β)	Odds Ratio	95% CI
Initial Weight (lbs)	0.011	1.01	1.01–1.02
Carbs (+10 g/day)	−0.067	0.94	0.91–0.97
Avg Number Final Logs	0.066	1.07	1.04–1.09

Note. Multivariate logistic model fit using variables selected with LASSO penalized regression. Dependent variable: Weight loss success (1 = ≥5% weight loss; 0 = <5% weight loss). N = 647 users meeting minimum 60-day food logging threshold. Model AIC = 805.11; AUC = 0.70. All VIF < 3, indicating acceptable multicollinearity. Odds ratios represent multiplicative change in odds for each unit increase in predictor. CI = confidence interval.

## Data Availability

The original contributions presented in this study are included in the article/Supplementary Material. Further inquiries can be directed to the corresponding authors.

## Supplementary Materials

The following supporting information can be downloaded at: https://www.mdpi.com/article/10.3390/nu18111766/s1. Table S1: Stratified Parsimonious Models Predicting Weight Loss Success. Figure S1: Calibration of final parsimonious model using predictors selected by LASSO.
