# Supplementary material for: Weight Loss Outcomes Among MyFitnessPal Users: Behavioral and Dietary Predictors of Success
Source: Nutrients. 2026 May 30;18(11):1766. doi: 10.3390/nu18111766 (PMC13258314; doi:10.3390/nu18111766)

Supplementary Table S1. Stratified Parsimonious Models Predicting Weight Loss Success

**Predictor**	**Estimate**	**OR**	**95% CI**
**Healthy Weight (BMI<25)**			
Initial Weight (lbs)	0.030	1.03	1.02-1.04
Carbs (+10g/day)	-0.102	0.90	0.85-0.96
**Overweight/Obese (BMI>=25)**			
Initial Weight (lbs)	0.012	1.01	1.01-1.02
Carbs (+10g/day)	-0.050	0.95	0.92-0.99
Avg Number Final Logs	0.070	1.07	1.04-1.11

Supplementary Figure S1. Calibration of final parsimonious model using predictors selected by LASSO.

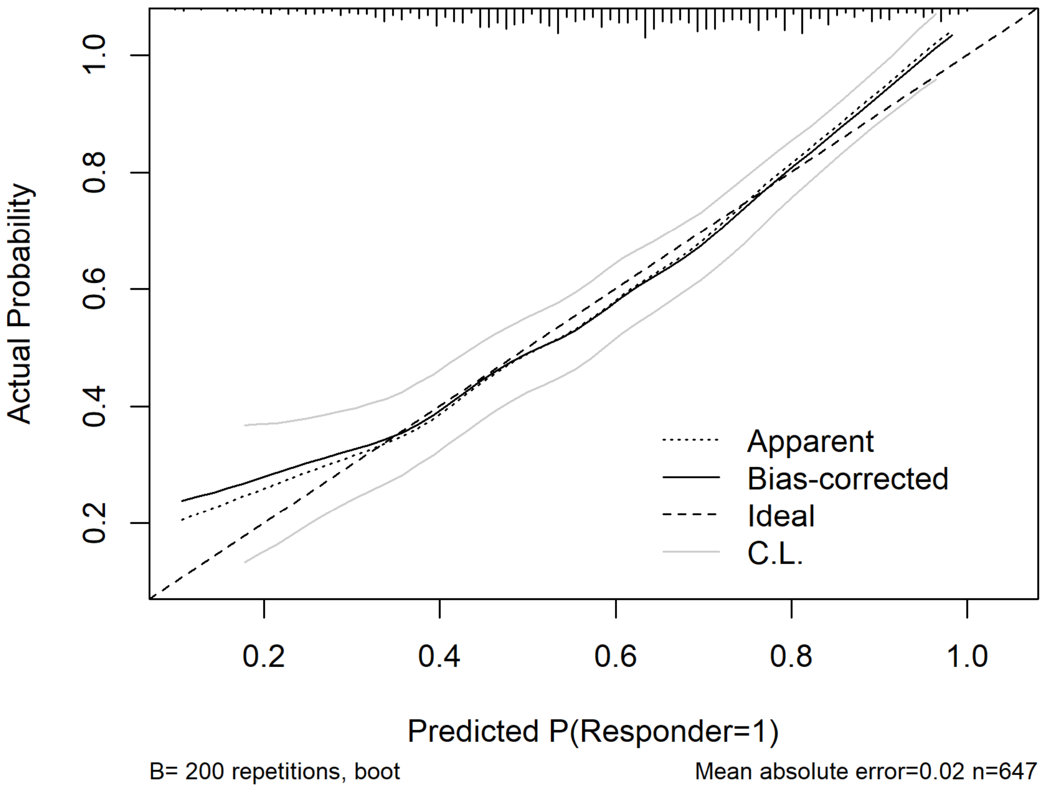

Supplement: Supplementary file 1 [file nutrients-18-01766-s001.zip › nutrients-4260166-supplementary.pdf]
